# Cases Illustrative of the Different Methods of Operating for the Stone

**Published:** 1826-09

**Authors:** M. Dupuytren, M. Sanson


### LITHOTOMY.

### *Cases illustrative of the different Methods of Operating for the Stone.* By MM. Dupuytren and Sanson.

A trial has recently been made at the Hôtel Dieu, in Paris, of the comparative merits of different methods of operating for the stone. M. Dupuytren undertook to perform the transverse or bilateral operation; M. Breschet, the lateral operation of Frère Côme; and M. Sanson adopted the recto-vesical operation, which has of late been very frequently employed in Italy by Vacca Berlinghieri. Each of these methods of operating were put in practice *alternately*, and therefore without any selection of cases.

The operation, as performed by M. Dupuytren, has been described by him in a Memoir laid before the Academie Royale de Medecine. The following is an abbreviated account of it:—The instruments required are a common staff, a bistoury, and a lithotôme, the form of which is nearly similar to that of Frère Côme, but it contains two curved blades, and these are made to separate in opposite directions by means of two levers (*bascules*), which are pressed against the handle of the instrument. The handle is moveable upon a screw, and so contrived that the separation of the two blades may be regulated at will to the extent of eighteen lines. The patient being placed and retained in the same posture as for the common lateral operation, a staff is introduced into the bladder, and held by an assistant in a vertical position. The operator, armed with a bistoury, makes a transverse incision, of half an inch in length, across the raphè, and about six or seven lines before the anus. This incision divides the skin and subcutaneous cellular texture, and has a slight curvature, the concavity of which presents backwards. A second incision divides the urethra at its inferior part, and the knife is then laid aside. Next, the extremity of the lithotôme is run along the groove of the staff into the interior of the bladder; and, the staff being removed, the lithotôme is so placed that the curvature may present its concavity downwards; the two levers are pressed against the handle of the instrument, which is withdrawn, dividing at the same time the neck of the bladder on either side of the median line, the two lateral lobes of the prostate, and a small portion of the membraneous part of the urethra. The rest of the operation has nothing particular in it: it is said to have the advantage of avoiding the essential parts of the generative organs, and the risk of wounding either the rectum or any large blood-vessel.

With regard to the method proposed by M. Sanson, it consists in cutting the inferior and anterior part of the rectum and the sphincter ani on the median line, and penetrating the bladder, either in dividing the neck of this organ surrounded by the prostate, or else its lower fundus. Dupuytren likewise has frequently practised this operation: he cuts as little as possible of the rectum, and makes use of a lithotôme, which he withdraws, cutting the prostate at the side about a line from the *veru montanum*, and without involving the vesiculæ seminales, although he does not think that their division would present any impediment to the generative functions. This method is exempt from the danger of hemorrhage, and the risk of inflammation is said to be less than in the other operations; so that, as regards the life of the patient, it is preferable: but it has the disadvantage of occasionally giving rise to fistulous communications between the rectum and bladder, which are very difficult of cure.

Neither the method nor the cases of M. Breschet appear to present circumstances of sufficient interest for insertion. We subjoin an account of four operations, by MM. Dupuytren and Sanson.

I. *Transverse Operation*, performed by M. Dupuytren.

—— Rowset, aged thirty-eight, of a lymphatico-sanguine temperament, and sufficiently robust in appearance, had experienced pain in making water since he was five or six years old. He was admitted at the Hôtel Dieu, the 14th of June, 1825, for incontinence of urine. M. Dupuytren introduced a sound into the bladder, and immediately discovered a calculus, situated near the neck of the organ: it appeared to be large and rough. The patient complained of pain at the extremity of the penis and along the course of the urethra, and of frequent hematuria, but always without gravel. For some years very severe suffering was experienced in the neighbourhood of the rectum: the pain came on in paroxysms, and the patient at such times was in the habit of introducing the fingers of the left hand into the anus; a practice which, he said, mitigated the violence of the agony, and which he therefore could not resist: the fore and middle fingers had, in consequence, become affected with whitlow.

From the day of his admission, he was prepared for the operation by frequent baths, gentle purgatives, refrigerant drinks, and moderate diet. During this interval, he was twice affected with retention of urine, and it was necessary to introduce a catheter. His face was flushed, and the thyroid gland had become considerably enlarged; a circumstance which was attributed to his struggles during the attacks of pain.

On the 1st of July, the operation was performed. The patient being placed in the usual position, a transverse incision was made with the point of a straight bistoury, seven or eight lines before the anus, having a curve, with the convexity presenting forwards. M. Dupuytren, being apprehensive lest the enormous dilatation of the lower part of the rectum, (caused by the repeated introduction of the fingers,) should expose this to injury from the blades of the lithotôme, introduced two fingers of the left hand into the gut, which he stretched in a transverse direction, and pushed back towards the coccyx. The first incision had divided the skin and cellular tissue, and the second cut through the more deep-seated parts down to the urethra. There was now considerable hemorrhage. An incision was made into the urethra with the bistoury, guided by the nail of the fore-finger, first backwards and then forwards, following the course of its axis. The double lithotôme was opened to No. 15, in consequence of the presumed size of the stone. This instrument divided the neck of the bladder, and the lateral parts of the prostate, to the right and left, and from behind forwards. There was a discharge of urine, the patient making continual expulsive efforts, and crying out violently. The forceps was introduced; the stone broke, and was extracted piecemeal: it was about the size of a hen's egg, and was apparently composed of uric acid. Three injections were thrown up. The hemorrhage continued so that about two pallets of blood were lost. M. Dupuytren had recourse to a plug in order to stop the bleeding. A canula, rolled round with linen and charpie, was introduced into the wound, and the apparatus retained by a T bandage. The patient was put to bed.

Immediately after the operation, the presence of the canula produced violent straining, which brought on the bleeding afresh, and clots of blood escaped between the lips of the wound and the envelop of the canula. At last, however, the patient was made to understand the danger of these efforts, and he remained quiet for some hours, when he again began to strain, in consequence of an urgent desire to make water: a clot of blood made its escape by the penis; the bladder, however, did not appear to be distended.

Next day, injections were thrown into the bladder through the canula. The day following, he had little pain, and but little fever, which soon disappeared altogether.

On the 7th of July, suppuration was established; and, on the 10th, the canula fell out spontaneously. For some days longer, the urine all passed by the wound; then half of it by the wound, and the other half by the urethra; and, finally, the whole flowed by the natural passage.

10th August, the patient was discharged without any complaint, and the wound almost entirely cicatrised.

II. *Transverse Operation*, by M. Dupuytren.

—— Scache, aged forty-six, of a sanguine temperament and good constitution, was admitted at the Hôtel Dieu, September 5th, 1825.

He had experienced pain in making water from the age of seven years. About twelve, he became subject to hæmaturia, and this disposition continued for several years: generally speaking, however, he only passed blood after exercise. At thirty, the pains returned; they were continued and acute. Soon after a calculus became impacted in the canal of the urethra, and was extracted by cutting upon it. After this the pains disappeared till he was forty-one, when he suffered afresh; having dysuria, the stream of urine being often suddenly interrupted, and loaded with mucus. From time to time little calculi were voided, with exacerbations of pain: from fifty to sixty were passed in the course of four years. Two months before his admission, he voided another, probably formed in the urethra; it was twenty-two lines in length, and four in circumference. At the same time, fetid gas occasionally made its escape by the urethra, with noise and erection of the penis. The noise was sometimes sufficient to wake his wife, who slept beside him. A month before his admission into hospital, he passed by the same canal a lumbricus worm, white, and eleven and a half inches long. These circumstances gave rise to a conjecture that there existed a communication between the bladder and rectum.

September 5th.—The pains continue; no hematuria; urine purulent and fetid; frequent desire to make water. The patient sounded by M. Dupuytren, who ascertained the presence of a calculus. The general health pretty good.

14th.—On the 12th and 13th, some preparatory measures were put in practice, as a dose of castor-oil, &c. To-day, at nine A.M.

he was sounded again; the perineum was shaved; the catheter, being introduced into the bladder, was held in a vertical position; the skin was stretched with the left hand of the operator, and the first incision, of a semi-eliptical shape, made, eight or ten lines from the anus. The lithotôme was opened to No. 12; the urine was evacuated; the stone, which was small, broke, and was removed at several times. No hemorrhage; two injections; and subsequently, the finger having been introduced into the bladder without discovering any thing, the patient was put to bed. Slight pain in the hypogastric region; and, an hour and a half after the operation, a fit of shivering, which lasted three-quarters of an hour. A few hours after, another rigor; continued pain; scanty sanguinolent oozing from the penis and wound. At six o'clock in the evening, headache not severe.

Venesection.

15th, in the morning.—Pulse regular, with little frequency; skin soft and warm; entire cessation of the pains in the hypogastric region. In the evening, some colic.

Bleeding to the extent of about two pallets. Emollient cataplasm to the lower belly.

17th.—The urine begins to flow in greater quantity by the urethra: its smell is still very fetid, and like that of feculent matter; its colour is yellowish, and it is mixed with purulent mucus.

The patient takes six pills a-day, composed of soft Venice Turpentine, gr. lx.; Acetate of Lead, gr. iv.; Extract of white Henbane, gr. vi.

Eight days after the use of these pills had been commenced, the urine was less fetid, and deposited less mucus; but colic soon came on, and was followed by purging. The pills were then discontinued. The wound gradually contracted, till only a few drops of fluid escaped by it. The pills were resumed, and continued till near the end of October, when the patient was discharged. The wound at this time was completely cicatrised, but the urine was still slightly catarrhal.

### III. *Recto-vesical Operation*, by M. Sanson.

Claude Varnet, aged nineteen, a taylor, of good constitution, but of a character habitually grave, was admitted at the Hôtel Dieu, October 4th, 1825. He complained of constant burning pain along the urethra, particularly at the extremity of the glans. These pains had begun only three years before, the patient having been previously affected for twelve months with acute pain about the anus and hypogastric region, extending to the kidneys. The urine flowed freely enough; the jet was never interrupted, and the general health little altered, although the patient was very thin, and reduced by chronic diarrhœa. On sounding, a calculus was detected, which appeared to be large.

After the usual preparation, the operation was performed by M. Sanson on the 10th. The staff *(catheter a ventre)* being introduced, a longitudinal incision was made on the median line of the

[illegible]

perineum, from behind forwards, which cut into the membranous portion of the canal. A probe-pointed bistoury was then introduced into this opening and the prostate; the neck of the bladder, and its inferior wall, divided to the extent of twelve lines above the anus: a large stone, of an oblong shape, was then extracted. No accident happened; there was no fever, and all the functions were regularly performed. On the tenth day, the wound made by the operation was cauterised. Notwithstanding this method, which was practised every day, it was only about the twentieth day that a little urine began to come by the penis, and even this improvement was transitory.

On the 20th of November, the cauterisation was discontinued; and the patient left the hospital on the 8th of December, unable to retain his urine or to pass it by the urethra. Since his discharge, M. Sanson has watched him, and it appears that latterly the wound has become smaller, and shown a disposition to heal.

IV. *Recto-vesical Operation,* by M. Sanson.

A boy, named Frederic, aged seven years, of lively disposition, had experienced pain about the penis in making water, and occasionally at other times, from his earliest infancy. He was admitted at the Hôtel Dieu, November 10th, 1825. At this time he had an acute and deep-seated pain in the abdomen, and he experienced a sensation of extreme smarting at the point of the penis, whenever he made water or took the slightest exercise. The stream was wont to be suddenly interrupted, and return again in the same manner. Other circumstances were favourable, and the operation was performed on the 15th. It proved tedious and laborious, and it was not without difficulty that the urethra was divided on the groove of the staff, (the cause of this difficulty is not mentioned.) M. Sanson introduced a bistoury, which was curved on the cutting edge, and divided the prostatic portion of the urethra, the neck of the bladder, and that part of the external sphincter which belongs to the rectum. A small pair of forceps was then introduced into the bladder, and a moderate-sized calculus, of blackish colour, extracted. The only inconvenience suffered by the patient was some slight pain in the belly, which speedily yielded to the application of leeches. On the sixth day, some clots of blood escaped by the wound, and some fears of hemorrhage were entertained; but these proved groundless. On the eleventh day from the operation, some urine passed by the penis: after this, a small quantity always flowed by the natural passage, but the urine did not all pass by the urethra for some time.

The patient was discharged perfectly cured on the 10th of December.

In the first of these operations, it will be observed that considerable hemorrhage took place; notwithstanding which, the patient did well. The bleeding which occurs during this operation is of little importance, unless it proceed from the division of some considerable artery. Sometimes it is even of use, as it diminishes the risk of inflammation by emptying the minute vessels, in which blood is apt to be accumulated by the irritation of the calculus.

Hemorrhage supervening to the operation is much more dangerous, and often follows that which is in appearance the most favourable. In fact, the division of the vessels would appear to produce a spasm, or retraction, which prevents the blood from flowing immediately; but, within an hour after, it not unfrequently happens that bleeding takes place, either externally or into the bladder. In the former case the evil is soon detected, but in the latter great attention is required. The following are the principal symptoms indicative of internal hemorrhage:—The patient experiences a sense of weight in the region of the bladder, and subsequently becomes sensible of its being distended; he has frequent desire to make water, but without being able to effect it; the face becomes pale, the pulse weak, and occasionally he falls into a state bordering on syncope, from which he only recovers on the accession of a fresh paroxysm of pain; if the hypogastric region be examined, a tumor will be perceived, formed by the distended bladder.

In either case it is necessary to have recourse to plugging, and this is to be done in the following manner:—A silver canula, three inches in length, five or six lines in diameter, and terminating at one end in a cul-de-sac, perforated by numerous holes, is to be procured: at some distance from this extremity, a *chemise* of fine linen is to be put on, and the canula introduced into the wound, till it arrives at the bladder; then between it and the linen, charpie is to be put in sufficient quantity to exert a degree of pressure capable of arresting the hemorrhage. The canula is to be secured by tapes, and attached to a bandage. If the bladder be already full, the surgeon should begin by introducing the finger, smeared with cerate, into the wound; and, in doing so, care would be required not to mistake for the bladder the little cul-de-sac which may be produced by pushing the finger against the recto-vesical partition. The finger being in the bladder, a syringe is to be guided so as to throw up injections; and care is to be taken to leave no clot; otherwise the bladder, contracting, will soon throw it out, and the canula along with it. To these means, which, though simple, are very efficacious, is to be attributed the favourable result of the case above alluded to.

With respect to the two examples of recto-vesical operation, it is to be observed that one of them gave rise to a fistula, probably owing to the extreme emaciation and weakness of the patient; while it is the opinion of the surgeon that this distressing infirmity was likely to be got rid of in time. The risk of a fistulous opening being left, is certainly the greatest objection to this form of operation; at the same time, there are numerous instances recorded, in which this has subsequently been healed. M. Dupuytren, having occasion to operate on an old man above eighty, gave the preference to this method, as least dangerous to life; besides which, the stone was large. A fistula followed, but it healed spontaneously a year after. M. Sanson practised this operation upon a young man, in a Maison de Santé: the patient was discharged with a fistula, and went to an hospital to be treated for it. The means adopted were unavailing, but the fistula healed spontaneously soon after he had returned home. M. Dupuytren operated, in 1823, on a man forty-five years of age: a fistula followed; this was cured by cauterisation in the course of some months. In the same year, a child was thus operated upon at the Hôtel Dieu; had fistula: cauterisation was employed without success, and he returned home. At the end of four months, the wound healed spontaneously. In 1824, a child was admitted at the Hôtel Dieu for a recto-vesical fistula, which had followed the operation, and been present for a year: it involved the neck of the bladder, and a part of the membranous portion of the urethra. Half the urine escaped by the wound, whenever the bladder was emptied; besides this, there was a constant oozing, in consequence of which the urine became accumulated in the rectum, occasioning irritation and some degree of purging. Feculent matters and stercoraceous gas likewise passed into the bladder, which suffered, in its turn, from the presence of these foreign bodies. M. Dupuytren applied the nitrate of silver: this was done by taking a gum-elastic sound, and removing a portion at one side and near the extremity: into this the caustic was introduced, and fixed with thread. He thus cauterised only one particular point, avoiding the neighbouring parts. After two applications, a much smaller quantity of urine flowed by the wound, and the little patient was speedily cured.—These examples show that this unfortunate consequence of the recto-vesical operation may be frequently removed, but they at the same time show the frequency of its occurrence.

With regard to the different methods of operating, no general conclusions can be drawn from these cases: all the patients recovered; and it might be safely said, with regard to lithotomy, that its success depends much less upon the method, or even the individual skill of the operator, than upon the constitution of the patient, and the preparatory and subsequent treatment.*

* Répertoire générale d'Anatomie, &c.

